# Development of an Educational Website for Patients With Cancer and Preexisting Autoimmune Diseases Considering Immune Checkpoint Blockers: Usability and Acceptability Study

**DOI:** 10.2196/53443

**Published:** 2024-10-25

**Authors:** Maria A Lopez-Olivo, Maria E Suarez-Almazor, Gabrielle F Duhon, McKenna Cherry, Huifang Lu, Cassandra Calabrese, Mehmet Altan, Hussain Tawbi, Alexa Meara, Clifton O Bingham, Adi Diab, Viola B Leal, Robert J Volk

**Affiliations:** 1 Department of Health Services Research MD Anderson Cancer Center The University of Texas Houston, TX United States; 2 Section of Rheumatology and Clinical Immunology of the Department of General Internal Medicine MD Anderson Cancer Center The University of Texas Houston, TX United States; 3 OCHIN, Inc Portland, OR United States; 4 Texas Woman's University Houston, TX United States; 5 Department of Rheumatologic & Immunologic Disease Cleveland Clinic Cleveland, TX United States; 6 Department of Thoracic-Head & Neck Medical Oncology MD Anderson Cancer Center The University of Texas Houston, TX United States; 7 Department of Melanoma Medical Oncology MD Anderson Cancer Center The University of Texas Houston, TX United States; 8 Division of Internal Medicine Wexner Medical Center Ohio State University Columbus, OH United States; 9 Division of Rheumatology Department of Medicine Johns Hopkins University Baltimore, MD United States

**Keywords:** immune checkpoint inhibitors, patient education, usability testing, cancer, autoimmune diseases, mobile phones, user testing, usability, user experience, immunotherapy, websites, development, acceptability, autoimmune, immunology, oncology, architecture, iterative, vasculitis, Crohn disease, Sjogren syndrome, educational, web-based resource, health information, rheumatology, arthritis, web design, eHealth, adverse events, patient care, treatment

## Abstract

**Background:**

Patients with cancer and an underlying autoimmune disease who are considering immune checkpoint blockers (ICBs) need to know about the benefits and risks of severe immune-related adverse events and flares of the autoimmune condition.

**Objective:**

This study aims to develop and alpha test an educational website for patients with cancer.

**Methods:**

Learning topics, images, and website architecture (including flow and requirements) were developed and iteratively reviewed by members of a community scientist program, a patient advisory group, and content experts. Alpha testing was performed, measuring the site’s usability using the Suitability Assessment of Materials and its acceptability using the Ottawa Acceptability Measure.

**Results:**

The website included a home page; general information about ICBs; comprehensive modules on the benefits and risks of ICBs for patients with cancer and preexisting autoimmune diseases; general wellness information; and features such as a quiz, additional resources, and a glossary. For the alpha testing, 9 users assessed the newly developed website. Patient reviewers (n=5) had rheumatoid arthritis, Crohn disease, Sjogren syndrome, or vasculitis. Health care provider reviewers (n=4) were medical oncologists or rheumatologists. The median Suitability Assessment of Materials rating was 75 (IQR 70-79; range 0-100) for patients versus 66 (IQR 57-72; range 0-100) for providers (scores ≥70 indicate no substantial changes needed). Recommendations for improvement, mostly involving navigation and accessibility, were addressed. All participants expressed that the website was acceptable and balanced in terms of discussion of benefits and harms. Because half (2/4, 50%) of the providers suggested we increase the amount of information, we extended the content on the impact of having an autoimmune disease when considering ICB treatment, the probability of flares, and the management of flares in this context.

**Conclusions:**

The feedback led to minor revisions to enhance readability, navigation, and accessibility, ensuring the website’s suitability as a decision-making aid. The newly developed website could become a supporting tool to facilitate patient-physician discussion regarding ICBs.

## Introduction

Health information can motivate patients to become involved in their health care and facilitate discussions between patients and health care providers [1]. Informed patients also have better treatment adherence and satisfaction with their care [2,3]. Although web-based information can be helpful, it can also be harmful as it may contain conflicting, biased, or incomplete information, causing confusion on the part of the patient. For instance, requests for interventions that may not be appropriate or may have unforeseen harms may emerge from unbalanced, poor-quality information that presents an overly positive picture [4].

Immune checkpoint blockers (ICBs) are immunotherapy drugs that enhance the immune system’s ability to target cancer by inhibiting specific pathways that regulate immune cell activity, such as programmed cell death protein 1, programmed death ligand 1, and cytotoxic T-lymphocyte–associated protein 4 [5]. These pathways are often exploited by cancer cells to evade immune detection. By blocking these checkpoints, ICBs unleash T-cells to attack cancer more effectively, significantly benefiting patients with various malignancies, including melanoma, nonsmall cell lung cancer, renal cell carcinoma, and others [6]. However, this approach can also trigger immune-related adverse events, as the heightened immune response may affect normal tissues [7].

Because ICBs have the potential to cause severe immune-related adverse events or exacerbate underlying autoimmune conditions, patients with cancer and a preexisting autoimmune disease who undergo treatment with ICBs need balanced information about ICBs [8-10]. Results from our prior learning needs assessment in this population suggest that some patients like to learn on their own to allow time to digest information and ask questions later [11]. We found that most patients preferred educational materials in multiple formats (eg, video, audio, graphics, and text), suggesting that websites or smartphone apps would be the most convenient delivery channels [11]. Clinicians also agreed that the optimal delivery of health information should include multiple formats; however, crucial requirements identified by providers were accuracy, simplicity, and standardized information (as opposed to individualized or nonlinear information) [12].

To our knowledge, there currently exists no web-based information containing specific content for patients with cancer and a preexisting autoimmune disease who are considering ICBs. Current websites provide only general information about cancer and ICBs, with few providing balanced information between benefits and potential risks [13]. We developed and alpha tested an educational website designed to inform patients with cancer and underlying autoimmune diseases who are considering ICBs and to facilitate patient-provider discussions.

## Methods

### Design

**Figure 1:**
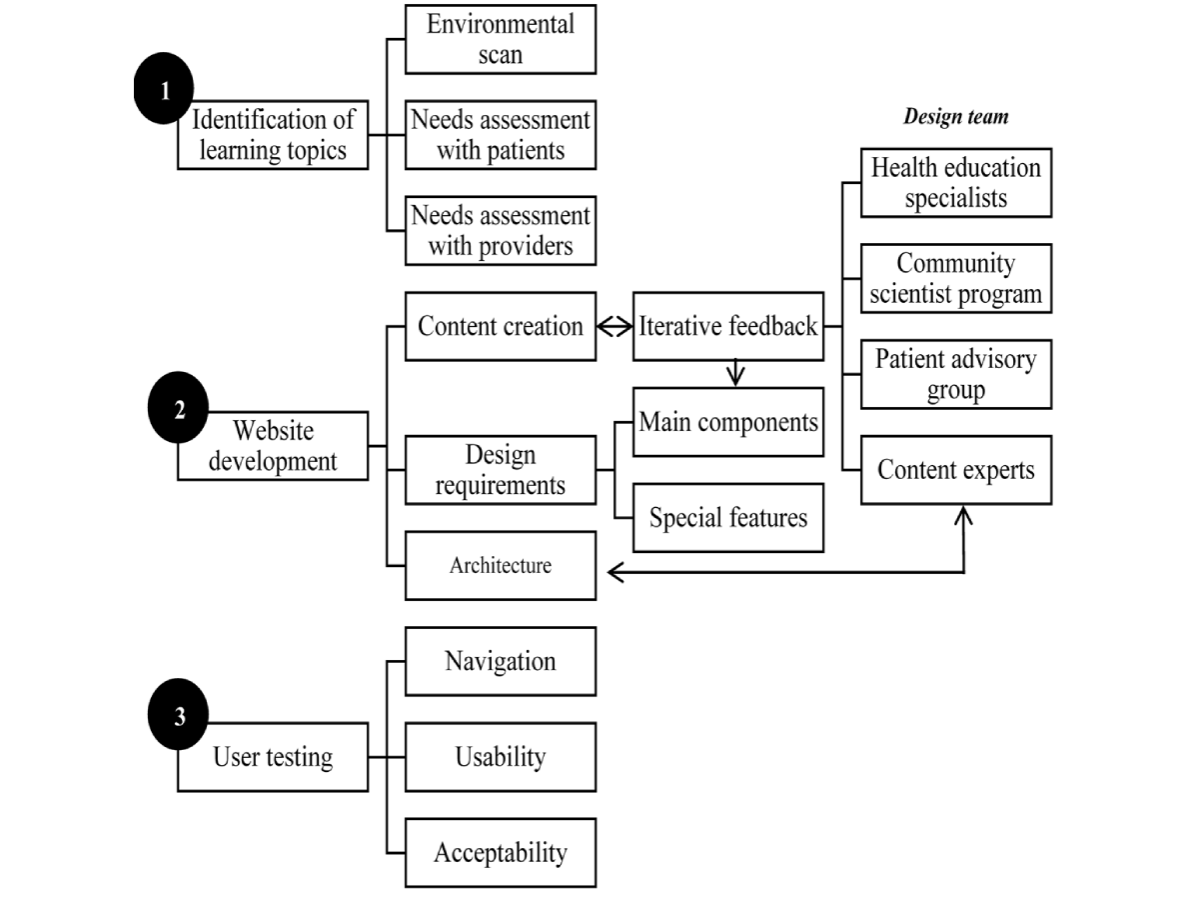
Study design.

We followed a user-centered approach to develop and test our website [14]. Our study process, depicted in Figure 1, involved 3 main sequential phases: identification of learning topics, website development, and user testing.

### Identification of Learning Topics

The process for identification of the key points to include in the educational content has been described elsewhere [12,13]. Briefly, an environmental scan was conducted to assess the quality and content of web-based information about ICBs [13]. Concurrently, we interviewed patients with cancer and a preexisting autoimmune disease who were considering or already had received ICBs, as well as the providers caring for these patients [11,12]. We asked both patients and providers about their preferred formats and channels to deliver information.

### Website Development

Two review authors (ME and MALO) created educational content based on the identified learning needs and current informational gaps. Two patient health education specialists helped to ensure that the content readability was at a sixth-grade level or below. We then focused on the website architecture and design requirements, including colors, layout, and text formatting. We identified the main components and special features to be included (ie, medical illustrations, glossary of terms, quiz based on the educational content, and links to other relevant URLs). For the mockup website, the informational components were categorized into basic, key, and other health-related information. A medical illustrator created visual representations of the concepts related to the immune system, immune cells, immune checkpoint proteins, and autoimmune diseases.

The learning topics; images that were relevant to the educational content; and website architecture, including flow and requirements, were iteratively reviewed by health education specialists, members of a community scientist program, a patient advisory group (ie, 3 patients who had received ICBs and 2 caregivers), and content experts (ie, 3 oncologists, 4 rheumatologists, and 1 decision scientist). Four Zoom (Zoom Video Communications, Inc) meetings were held with members of the community scientist program (an institutional resource to gain consumers’ input on research projects), which includes patients with cancer, survivors, and caregivers (the number of participants in each of these groups differed for every meeting, n=8-12). Moderators of the meetings took written notes, and this information was used to modify the website content. Feedback on the mockup website from the patient advisory group and the content experts was received through individual interviews and email communications, and a disposition report was created that summarized all comments and how they were addressed. Screenshots of the mockup website are shown in the Multimedia Appendix 1.

### Patient and Provider Testing of the Prototype

After developing the mockup website, we conducted an extensive evaluation of the prototype (alpha testing). Nielsen studies suggest that 5 users from any user group will elicit 80% of interface usability problems [15]. The purpose of the testing was primarily to assess visual elements, content of the website, navigation, functionality, acceptability, and usability.

### Recruitment

Participants were recruited from a large comprehensive cancer center. We posted flyers in participating clinics. In addition, research staff identified potentially eligible patients by reviewing clinic schedules and through a chart review. Patients met eligibility criteria if they were diagnosed with an underlying autoimmune disease, had already received ICBs, were fluent in English, and had access to a device with Zoom conferencing capabilities. Providers were eligible if they were medical oncologists or internal medicine specialists caring for these patients. Potential participants were contacted by phone, message, or email by a member of the research team and invited to participate.

### Ethical Considerations

The study was approved by the institutional review board at The University of Texas MD Anderson Cancer Center (protocol #2020-0843). All participants provided verbal consent to participate. Informed consent was obtained from all participants involved in the study. Members of the patient advisory board and patients completing the alpha testing were compensated ($180 gift card for members of the advisory board and $30 for patients). Study data were collected and managed using a secure, web-based software platform, REDCap (Research Electronic Data Capture; Vanderbilt University) hosted at The University of Texas MD Anderson Cancer Center [16,17].

### Procedures

After providing informed consent, participants navigated the website using computers connected to the internet at their homes using Zoom or in the clinic on mobile devices. Two investigators (MALO and GFD or VT) led the user testing session, with MALO sitting in on most sessions to take notes. We used 3 different approaches for testing as follows.

- Navigation testing: During cognitive interviews, we asked participants to describe how they were navigating through the website [18]. Written notes were taken of participant reactions and behaviors. We asked questions about the general look and feel of the mockup website (eg, language and terminology, information layout, and images), menu options and pathways, and how they would normally access the website (smartphone or PC). We instructed participants to complete some basic tasks to find information on the website to test its functionality. In addition, we asked about the appropriateness of the content and images (see Section S1 inMultimedia Appendix 2). Navigation sessions lasted between 52 and 64 minutes for patients and 30 to 60 minutes for providers. They were conducted between March 2023 and July 2023.
- Usability: We used the Suitability Assessment of Materials (SAM) to evaluate the adequacy of the content, literacy demand, graphics, layout and typography, learning stimulation and motivation, and cultural appropriateness [19]. Each item in SAM has 3 possible responses: “not adequate,” “adequate,” and “superior.” A “superior” response scores 2 points for that item; an “adequate” response scores 1 point; and a “not adequate” response scores 0 points. These points are then added up and divided by the maximum possible total score (ie, 44) to obtain a percentage rating (hereafter referred to as the SAM score). A SAM score of 70 to 100 is considered superior with no need for revisions. A SAM score of 40 to 60 is considered suitable, but revisions may be needed for any items considered unsuitable. A SAM score of less than 40 is considered not suitable [20,21].
- Acceptability: The Ottawa Acceptability Measure was used to obtain patient ratings of various features of the educational tool, including length and amount of information, type of information (balanced or not), and likelihood to help people with cancer and autoimmune diseases who are considering ICBs. Responses can be reported descriptively in terms of proportions responding positively or negatively on each criterion. The scale also includes an open-ended question about the overall satisfaction with the website [22].

Other measures collected included patient demographics (ie, age, sex, race, ethnicity, language, and education), health literacy using the Single Item Literacy Screener to identify patients who have difficulty reading health-related materials [23], and preferred decision-making role (the patient is the primary decision maker [active], the provider is the primary decision-maker [passive], or the provider and patient make the decision together [collaborative]) using the Control Preferences Scale [24].

### Analysis

We used a mixed methods approach for data analysis. Notes from the navigation testing with participants and the responses to the Ottawa Acceptability Measure open-ended questions were collated and categorized into themes related to acceptability, usability, accessibility, navigation, and functionality of the website. Changes were made if more than 2 patients or providers suggested areas of improvement. We used descriptive statistics to summarize the characteristics of the participants and the data from the usability and acceptability scales.

## Results

### Website Components

**Figure 2:**
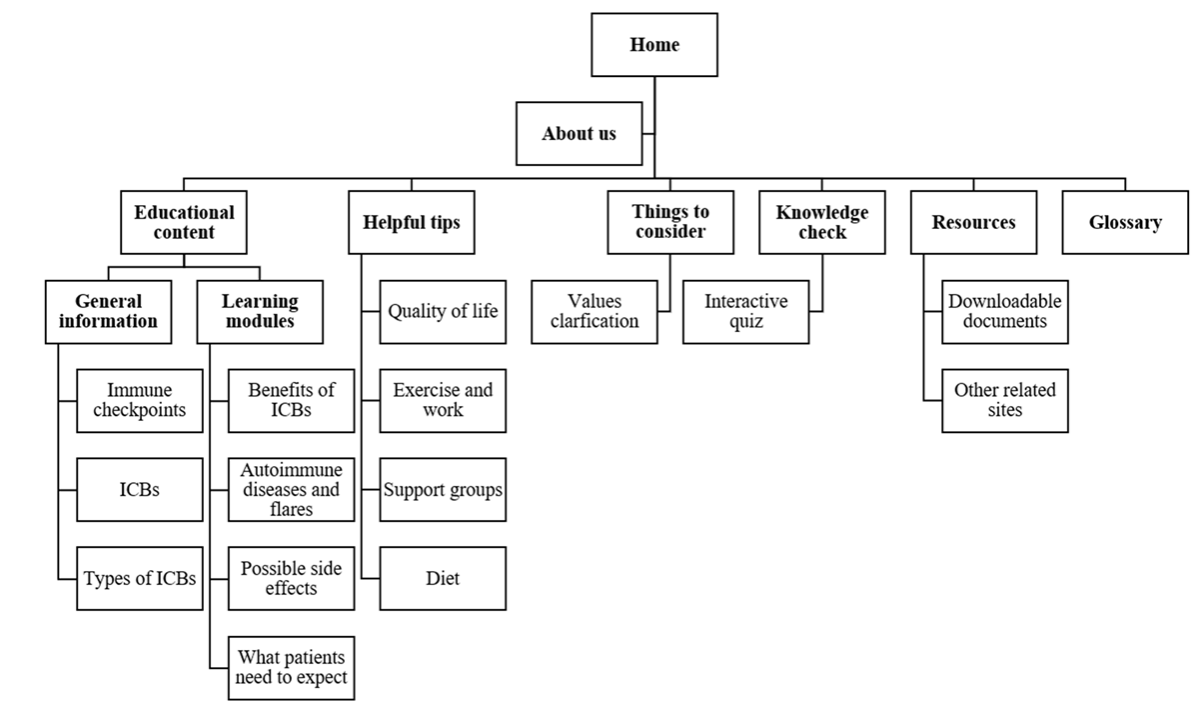
Site map for the newly developed website. ICB: immune checkpoint blocker.

Figure 2 shows the components included in the website. The final site map includes a page with general information about ICBs (ie, “What cancer is?” “What the immune system is?” “What T-cells are?” “How the immune system responds to cancer?” “What immunotherapy is?” “What immune checkpoints are?” ICB mechanisms of action, and types of ICBs) and learning modules covering (1) benefits of ICBs (ie, choice of treatment, benefits of ICBs, ICB treatment vs other cancer treatments, and ICB therapy vs chemotherapy); (2) receipt of ICBs in the context of autoimmune disease (ie, “What autoimmune diseases are?” “What flares are?” “How can ICBs affect autoimmune diseases?” importance of autoimmune disease control when considering ICB treatment, risk of flares during ICB treatment, possible symptoms, treatment options, and appointment with autoimmune disease specialist); (3) possible side effects (ie, side effects that are not flares of the autoimmune disease, symptoms that require immediate attention, permanent or fatal side effects, and when to call a doctor); and (4) what patients should expect before, during, and after treatment with ICBs (ie, discussion with doctor, tumor markers, ICB treatment process, length of ICB treatment, ICB monotherapy vs combination, ICB combined with other cancer treatments, and causes of ICB discontinuation). An additional page includes information about the potential impact of ICBs on quality of life as well as exercise and daily activities, support groups, and maintaining a healthy diet. The site also includes a quiz, a values clarification booklet with possible questions to ask doctors, a glossary page with definitions of the medical terminology used throughout the site, and a resources page with links to downloadable documents and related sites. The “About Us” page includes the names and affiliations of those involved in the development and production of the website, a health information disclaimer, and the sources of funding.

### Website Maintenance

The website displays an update date to inform users of the most recent revisions. To ensure the website remains up-to-date and relevant, the content will be reviewed and updated every 24 months or more frequently as new evidence emerges. Updates will be managed by the research team, which maintains a database of relevant medical literature on the topic [25,26]. Specific updates will include incorporating new ICB indications, advances in toxicity management, and any critical findings relevant to autoimmune disease risks.

### Patient and Provider Testing of the Prototype

Patient participants (n=5) had a mean age of 59.2 (SD 11.6) years; 3 (60%) were female, 2 (40%) had diagnosis of rheumatoid arthritis, 1 (20%) had Crohn disease, 1 (20%) had Sjogren syndrome, and 1 (20%) had granulomatosis with polyangiitis. The following malignancies reported were each reported once (n=1, 20%): lung cancer, prostate cancer, melanoma, colon cancer, and breast cancer. The ICB administered, education level, race, ethnicity, health literacy, and preferred decision-making role of the participants are summarized in Table 1.

**Table 1:** Characteristics of patient participants (n=5).

Characteristic			Values
**Age (years), mean (SD)**			59.2 (11.6)
**Sex, n (%)**			
	Female	3 (60)	
**Education level, n (%)**			
	Less than high school diploma	1 (20)	
	High school diploma or higher degree	4 (80)	
**Race or ethnicity, n (%)**			
	Hispanic White	1 (20)	
	Non-Hispanic White	4 (80)	
**Health literacy, n (%)**			
	Adequate	3 (60)	
	Inadequate	2 (40)	
**Preferred decision-making role** **, n (%)**			
	Collaborative	4 (80)	
	Active	1 (20)	
**Immune checkpoint blocker administered** **, n (%)**			
	Durvalumab	2 (40)	
	Nivolumab	2 (40)	
	Pembrolizumab	1 (20)	

Provider participants (n=5) comprised 4 (80%) melanoma medical oncologists and 1 (20%) rheumatologist. Three (60%) were female, 3 (60%) were White, and 2 (40%) were Asian, with a median of 18 (IQR 16-20) years of practice. The median SAM score for patient participants was 75 (IQR 70-79; range 0-100). For provider participants, the median SAM score was 66 (IQR 57-72; range 0-100). Providers reported seeing an average of 20-200 patients who are receiving ICBs, and providers spent 20% to 75% of their time in clinical practice. All reported being confident in managing patients with cancer and preexisting autoimmune diseases.

### Usability

The median SAM score for patient participants was 75 (IQR 9.1; range 0-100). For provider participants, the median SAM score was 64 (IQR 14.2; range 0-100). The number of patients and providers rating specific items on SAM as adequate or superior is shown in Table 2 [19]. Only 2 items were considered for revision: consistently providing context before presenting new information and adding step-by-step directions for the interpretation of medical illustrations used. We did not consider making changes to the typography because our text met the criteria for suitability (ie, consistent use of upper and lower case with serif font type, font size of at least 12 points, bolding and change of color, and size used to emphasize key points).

**Table 2:** Proportion of participants rating each item on the Suitability Assessment of Materials as adequate or superior.

Item		Patients (n=5), n (%)	Providers (n=4^a^), n (%)
**Content subscale**			
	Purpose is evident	5 (100)	4 (100)
	Content	5 (100)	4 (100)
	Scope is limited	5 (100)	4 (100)
	Summary of review included	5 (100)	4 (100)
**Literacy demand subscale**			
	Reading grade level	5 (100)	3 (75)
	Writing style, active voice	5 (100)	4 (100)
	Vocabulary with common words	5 (100)	3 (75)
	Context given first	4 (80)	4 (100)
	Learning aids via “road signs”	5 (100)	4 (100)
**Graphics subscale**			
	Cover graphic showing purpose	5 (100)	3 (75)
	Type of graphics	5 (100)	4 (100)
	Relevance of illustrations	5 (100)	3 (75)
	Lists and tables explained	4 (80)	3 (75)
	Captions used for graphics	5 (100)	4 (100)
**Layout and typography subscale**			
	Layout easy to follow	5 (100)	4 (100)
	Typography appropriate	4 (80)	4 (100)
	Subheading “chunking” used	5 (100)	4 (100)
**Learning stimulation and motivation subscale**			
	Interaction used	5 (100)	4 (100)
	Behaviors modeled and specific	5 (100)	4 (100)
	Motivation and self-efficacy	5 (100)	4 (100)
**Cultural appropriateness subscale**			
	Match in logic, language, experience	5 (100)	4 (100)
	Cultural image and examples	5 (100)	4 (100)

^a^One clinician provided only verbal suggestions after navigation.

### Acceptability

Patient participants agreed that the website was acceptable, with good or excellent information regarding the impact of preexisting autoimmune diseases in the context of ICB therapy and risk of flares (Table 3). Patients perceived the information as balanced (benefits or harms ratio) and containing enough information to be helpful for making a decision regarding the use of ICBs (Table 3). Providers were neutral about the length of information, preferring more information in general, especially about treatment options. As a result of this feedback, we expanded the amount of information provided about treatment options, ICB infusions, and disease flares of underlying autoimmune diseases.

**Table 3:** Proportion of participants rating each item on the Ottawa Acceptability Measure as good or excellent.

Ottawa Acceptability Measure item			Patients (n=5), n (%)		Providers (n=4^a^), n (%)
**Type of information presented**					
	Impact of preexisting autoimmune disease	5 (100)		2 (50)	
	Risk of flares	4 (80)		2 (50)	
	Treatment options	3 (60)		2 (50)	
	Right length of information	4 (80)		2 (50)	
	Right amount of information	4 (80)		2 (50)	
	Balanced information	5 (100)		4 (100)	
	Educational tool useful at the time of first discussing treatment with immune checkpoint inhibitors	5 (100)		4 (100)	
	Enough information to help patients decide whether to use immune checkpoint inhibitors	5 (100)		3 (75)	

^a^One clinician provided only verbal suggestions after navigation.

### Qualitative Synthesis of Suggestions

Patient participants appreciated the ICB overview. Most comments were favorable; patients expressed that having this website before starting ICB therapy would have made it easier to understand the information and their decisions. One participant commented, *“*It was really informative. I wish I had it to know what I was getting into.” Another commented*,* “I was able to read it. I like it was in common words that I could understand.” Other participants commented on the amount of information. For example, a participant stated, “I think it was just right what was in there. Not too much, not too little. It gave enough info to decide.” Others commented on the additional pages, such as the glossary of terms and features of the site (“crisp” images, breakdown of the content, and ability to move from 1 module to another).

Table S1 in Multimedia Appendix 2 contains a summary of the suggested website changes. Recommendations for improvement mostly involved minor changes to the content to improve readability and expand information to include more details about the immune system and autoimmune diseases and treatment. Other recommendations involved navigation (eg, adding a home page button and site map), accessibility (eg, enlarging images and including activities), and functionality (eg, adding a link to the institutional patient portal for direct messaging to the clinic).

## Discussion

### Principal Findings

This paper details the design, development, and evaluation of an educational website with information tailored for patients with cancer and preexisting autoimmune diseases considering treatment with ICBs. The website included comprehensive information on ICBs, their benefits and risks, and the implications for patients with autoimmune conditions, alongside tools such as a quiz and a glossary. Both patient and provider participants found the website usable and acceptable. Patients appreciated the clarity and relevance of the content, while providers suggested expanding information on treatment options. Minor improvements were recommended to enhance readability, navigation, and functionality, indicating the website’s potential as a valuable resource for informed decision-making in this patient population.

In the United States, more than 1 in 3 individuals currently access web-based health information [27], and its use has grown substantially over the past decade [28,29]. Therefore, the creation of a dedicated website for patients with cancer and preexisting autoimmune disease appears to be a cost-effective and widely accessible solution to address some of the unmet informational needs of this patient population [11]. Reliable web-based information can complement the role of clinicians in health education, making web-based information a valuable supportive tool in the overall health care process [30-32]. Furthermore, web-based information platforms can play a crucial role in supporting individuals in remote areas and those without access to health care education services. In clinical settings with vast demand and insufficient time to allocate to ensure that patients comprehend the information received by the health care team, web-based information can also play an important role.

Relying on Google or other search engines for health information can cause problems when the information available is inaccurate, of low quality, not relevant to the individual’s needs, or inconsistent across various sources. In fact, such poor information may have negative effects on communication between patients and health care providers [30,31]. In our previous research, patients expressed a desire for trustworthy sources of information that align with their health care provider’s recommendations. Similarly, health care providers welcomed patients sharing web-based health information if it was accurate and relevant to their medical needs [11,12].

Based on the previous research findings, we designed our website to address the specific needs of patients with cancer and preexisting autoimmune diseases. Our research indicated that these patients face challenges in obtaining comprehensive and reliable information about ICBs and the potential for immune-related adverse events and autoimmune disease flares [11,13]. Both patients and clinicians expressed a desire for a trustworthy and easily accessible source of evidence-based information, which our website aims to provide [12,13]. Our past research has also shown that although patients are given information about immune-related adverse events and flares from their clinicians at the time of the first encounter, patients often fail to understand or remember it [11]. In addition, current information about ICBs is not specific for patients with preexisting autoimmune disease, thus clinicians cannot offer resources with specific information for patients to review after their encounter [13].

During the design and development phases, the research team recommended creating a user-friendly site with content that met the plain language guidelines for patients with low health literacy, in addition to presenting high-quality, evidence-based information. The objectives were achieved successfully because the design, content, illustrations, and language used were well received by the users. The prototype testing revealed that the website was user-friendly and easy to navigate. Additionally, participants found the content highly suitable for their needs.

A strength of our study was the adoption of a user-centered design approach, effectively addressing users’ requirements for a website that was easy to use, comprehensive, evidence-based, and backed by reputable experts and health care providers. Another strength was the engagement of a diverse group of multidisciplinary expert clinical advisors and a design team with extensive knowledge and experience in user-centered design.

As with any research study, ours also had some limitations. First, our team has limited representation of participants with low health literacy, who may have provided different feedback than that from our participants. However, we addressed this by including health literacy experts among the production group. Second, for assessing the website’s usability and acceptability, patients were deliberately selected from individuals who had already received ICBs to ensure an in-depth experience. However, it is important to acknowledge that this sampling approach may not fully represent the feedback that we could have received from individuals who have not yet been exposed to ICBs. Nonetheless, the recommendations collected closely aligned with those from our patient advisory board and the community scientist program, both of which include individuals without previous knowledge of immunotherapy or autoimmune diseases. Finally, the type of web-based platform chosen to address users’ needs was predetermined before the study began because previous research indicated a preference for channels that would allow delivery of the information in multiple formats [33]. It is possible that a smartphone app or the electronic health record system could have been used to deliver text and illustrations and include interactions but the chosen format was based on our preliminary work [11,12].

Ongoing data collection will provide more information to evaluate the effectiveness of the newly developed website in enhancing knowledge, facilitating patient-provider discussions, and continuing to meet the end users’ needs. Additionally, further research is planned to enhance specific sections of the website, incorporating more complex features to address users’ requests. To improve accessibility, we also aim to translate the content into other languages.

### Conclusions

We used a human-centered design approach, involving the user throughout the design process to ensure that the final website met the needs and requirements of the targeted population. The research team, encompassing multiple stakeholders (ie, patients, caregivers, educators, physicians, and researchers), was involved throughout the design process. Our newly developed website was acceptable for patients and has the potential to become a supporting tool to facilitate patient-provider discussions regarding ICBs.

## Appendix

Multimedia Appendix 1 Screenshots of the mockup website.

Multimedia Appendix 2 Interview guides and website design recommendations.

## Abbreviations

ICB: immune checkpoint blocker
REDCap: Research Electronic Data Capture;
SAM: Suitability Assessment of Materials

## Data Availability

The datasets generated during and/or analyzed during this study are available from the corresponding author on reasonable request.
