# Supplementary material for: Development of an Educational Website for Patients With Cancer and Preexisting Autoimmune Diseases Considering Immune Checkpoint Blockers: Usability and Acceptability Study
Source: JMIR Cancer. 2024 Oct 25;10:e53443. doi: 10.2196/53443 (PMC11549586; doi:10.2196/53443)

**Multimedia Appendix 1.** Screenshots of website with educational content for patients with cancer and a preexisting autoimmune disease considering immune checkpoint inhibitors.


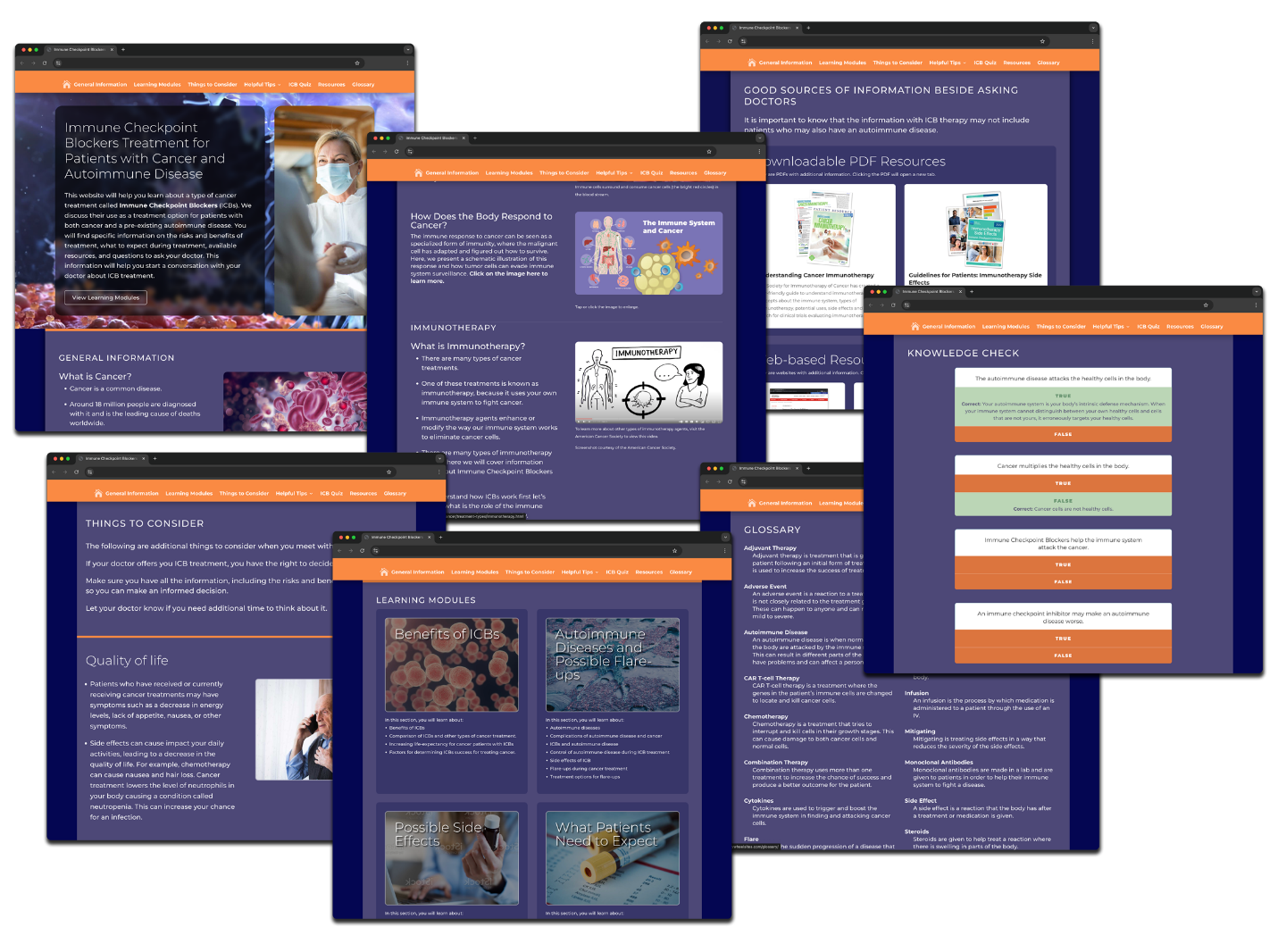

Supplement: Multimedia Appendix 1 [file cancer_v10i1e53443_app1.docx]
