# Supplementary material for: Development of an Educational Website for Patients With Cancer and Preexisting Autoimmune Diseases Considering Immune Checkpoint Blockers: Usability and Acceptability Study
Source: JMIR Cancer. 2024 Oct 25;10:e53443. doi: 10.2196/53443 (PMC11549586; doi:10.2196/53443)
Supplement: Multimedia Appendix 2 [file cancer_v10i1e53443_app2.docx]

## **Multimedia Appendix 2**

**Section S1.** Interview guides.

**Questions for providers**

**TASK 1**

This is a website we made with information about immune checkpoint inhibitors. We would like your opinion about the content and layout. I want you to explore this website. Look around at the information and navigate freely. Tell me when you think you are done.

RESEARCH STAFF: Write notes (participant reactions, behavior) per web page.

___________________________________________________________________________

___________________________________________________________________________

- What do you think about the design and layout of this website? Please explain. ________________________________________________________________

__________________________________________________________________

Notes/ Additional Probes:

*Take notes about the behaviors at each tab.* ________________________________________________________________________

________________________________________________________________________

**TASK 2**

I’m going to ask you to do some tasks on the website. The goal here is to get an idea if the script and graphics are understandable. I'd like you to think aloud as we go. As you look at the graphics and read through the text, just tell me everything you are thinking. At times I will stop and ask you more questions about the terms or phrases used in the narration. I will be also taking notes at the same time. Please keep in mind that I really want to hear all of your opinions and reactions. Don't hesitate to speak up whenever something seems unclear or is hard to answer.

**Section #** Please review the content about [pipe each website section].

*Reading through the section, do you agree with the content?*

*Is there anything that is unclear or inaccurate? Please explain.*

*Is it relevant?*

*Would you use this with your patients? If so, how so?*

*Can it be improved? Please explain.*

*Does the image reflect the content?*

Notes/ Additional Probes for each slide:

_____________________________________________________________________

________________________________________________________________________

**That concludes the navigation portion of our interview. We now would like to know if this will be useful to your patients and your practice, and if you have any suggestions for improvement.**

When would you want to give this educational content to patients?

How would you deliver this educational content to your patients within your clinical workflow?

Do you have any ideas or suggestions for improvements?

__________________________________________________________________

__________________________________________________________________

Do you have any final thoughts, suggestions, or feedback?

We have reached the end of the study. Thank you for your contributions.

**Questions for patients**

**TASK 1**

This is a website we made with information about immune checkpoint inhibitors. I want you to explore this website. Look around at the information. Tell me when you think you are done.

RESEARCH STAFF: Write notes (participant reactions, behavior) per web page.

___________________________________________________________________________

___________________________________________________________________________

- What do you think about the design and layout of this website? Please explain. ________________________________________________________________

__________________________________________________________________

Please look around at the information and navigate freely. I want you to tell me what you are doing as you are doing it. As you look at the material, try to tell me where you are going and why.

**Notes/ Additional Probes:**

*Take notes about the behaviors at each tab.* ________________________________________________________________________

________________________________________________________________________

Questions to consider:

- - I noticed you didn’t [probes: scroll down/click on]. Can you tell me why?

__________________________________________________________________

__________________________________________________________________

- - Did you notice the images about “_______?”

__________________________________________________________________

__________________________________________________________________

- - Can you tell me in your own words what this information [or this term] means to you?

__________________________________________________________________

__________________________________________________________________

- - Was this part easy or difficult to understand? How so?

__________________________________________________________________

__________________________________________________________________

- - Do you have any ideas or suggestions for improvements?

_________________________________________________________________

__________________________________________________________________

**TASK 2**

I’m going to ask you to do some tasks on the website. At times I might ask you to explain what you did. Tell me when you think you are done with the task.

How would you go about finding the (**Section #** Please review the content about [pipe each website section]).

Did the participant successfully complete the task?

Yes

No

Time on task: ____________

*Take note of the steps taken to complete task*

Notes:

________________________________________________________________________

________________________________________________________________________

That concludes the navigation portion of our interview. Do you have any additional thoughts, suggestions, or feedback?

**Table S1.** Website design recommendations.

| **Suggestion** | **Made by** | **Website changes** |
| --- | --- | --- |
| Content |  |  |
| Incidence (percentages) of side effects specific to each autoimmune disease should be added | Patient | Not possible because data are available for only some autoimmune diseases |
| Survival (percentages) of those who have received immunotherapy should be added | Patient | Plan to incorporate in second version |
| Scope statement should be longer | Patient | Revised to read: “This website will help you learn about a type of cancer treatment called immune checkpoint inhibitors (ICIs). We discuss their use as a treatment option for patients with both cancer and a pre-existing autoimmune disease. You will find specific information on the risks and benefits of treatment, what to expect during treatment, available resources, and questions to ask your doctor. This information will help you start a conversation with your doctor about ICI treatment.” |
| Introduction on immunotherapies in general should be added | Patient | The general information section was expanded to cover two topics: “immune system” and “immunotherapies” |
| More information on quality of life, diet, and support groups should be added | Patient,  provider | Sections expanded to include links to institutional resources on each topic |
| Types of immune checkpoint inhibitors should be presented as a list | Patient | Added a table listing agents currently approved by the US Food and Drug Administration |
| More general information about autoimmune disease and specific autoimmune disease treatments should be added | Patient | Added general information about immune system and autoimmune disease |
| Features (functionality) |  |  |
| Glossary should be linked to the institution’s glossary and terms included in medical illustrations should be added | Patient | Added as requested |
| A link to MyChart should be included for direct messaging to the clinic | Patient | Plan to incorporate in second version |
| Navigation |  |  |
| “About this website” should be a separate page | Patient,  provider | Separated as suggested |
| Top bar needs improvement to stand out, and drop-down menu should be removed | Patient | Tabs in the top bar were modified with different font color and size; dropdown submenus were eliminated |
| A site map should be added | Patient,  provider | Due to the simplicity of the website, it was recommended to not include a site map for this version |
| Sliders should have an intuitive start and end | Patient,  provider | Slider thumbnails will be added to eliminate overlap |
| Should be more intuitive how to close the sliders in the learning modules (struggled to get back to page) | Patient, provider | Close button for slider is now closer to the slider overlay |
| Accessibility |  |  |
| Images in the modules should be clickable | Patient,  provider | Images in the general information module can be zoomed in and out; images in the sliders were divided and enlarged |
| More interaction should be included | Patient | Added an interactive quiz, and an interactive risk calculator is under development for the second version |
| Should be more obvious that links are clickable | Patient | A button labeled “click here to access…” was added to the text |
| Because “portions of this will probably go over the head of the lay public,” images should be very practically driven so that patients visit the website for answers to their “problems” | Provider | Medical illustrations were modified to enhance clarity, and a guiding note was added for easy interpretation of images describing information at a cellular level |
